# In situ friction study of Ag Underpotential deposition (UPD) on Au(111) in aqueous electrolyte

**DOI:** 10.1002/cphc.202100130

**Published:** 2021-05-04

**Authors:** Inhee Park, H. Baltruschat

**Affiliations:** ^1^ Institut für physikalische und Theoretische Chemie Universität Bonn Römerstraße 164 53117 Bonn Germany; ^2^ Institut für physikalische und Theoretische Chemie Universität Bonn Römerstraße 164 D-53117 Bonn Germany

**Keywords:** Ag underpotential deposition, lateral force microscopy, adsorbate coverage, friction, tribology

## Abstract

The electrodeposition of silver on Au(111) was investigated using lateral force microscopy (LFM) in Ag^+^ containing sulfuric acid. Friction force images show that adsorbed sulfate forms $\left(\sqrt{3}\times \sqrt{7}R19.1^{\circ }\right)$ structure ($\theta_{sulfate}=0.2)$ on Au(111) prior to Ag underpotential deposition (UPD) and $\left(\sqrt{3}\times \sqrt{3}R30^{\circ }\right)$ structure ($\theta_{sulfate}=0.33$ ) on a complete monolayer or bilayer of Ag. Variation of friction with normal load shows a non‐monotonous dependence, which is caused by increasing penetration of the tip into the sulfate adlayer. In addition, the friction force is influenced by the varying coverage and mobility of Ag atoms on the surface. Before Ag coverage reaches the critical value, the deposited silver atoms may be mobile enough to be dragged by the movement of AFM tip. Possible penetration of the tip into the UPD layer at very high loads is discussed as a model for self‐healing wear. However, when the coverage of Ag is close to 1, the deposited Ag atoms are tight enough to resist the influence of the AFM tip and the tip penetrates only into the sulfate adlayer.

## Graphical abstract

**Ag underpotential deposition (UPD) on Au(111)** is investigated using lateral force microscopy (LFM). Variation of friction with potential shows that the coverage of adsorbate layers (Ag and sulfate) has a great influence on the friction. The friction forces plotted as function of load demonstrate once again a non‐monotonic dependence of the friction force on the adsorbate (sulfate and Ag) coverage, which results from the penetration of the AFM tip into adsorbate layers.

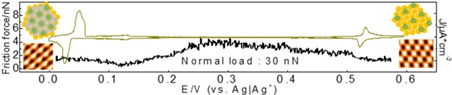

## 1 Introduction

Silver electrodeposition on Au(111) has been the subject of numerous studies because the interaction between foreign metal and substrate is very strong and the lattice mismatch is negligible.[^1^, ^2^, ^3^, ^4^, ^5^, ^6^, ^7^, ^8^, ^9^] The Ag UPD occurs in at least three different steps on Au(111) in sulfuric acid. Itaya et al. and Kolb et al. reported the structures of surface during Ag UPD in sulfuric acid using STM.[^1^, ^2^, ^3^, ^8^] They observed the $\left(\sqrt{3}\times \sqrt{7}R19.1^{\circ }\right)$ structure at the most positive potential, which indicates that sulfate/bisulfate is adsorbed, and forms ordered structure. At a potential negative of the 1^st^ Ag UPD peak, Itaya and coworkers observed a $\left(\sqrt{3}\times \sqrt{3}R30^{\circ }\right)$ structure but Kolb and coworkers observed a $\left(\sqrt{3}\times \sqrt{3}R30^{\circ }\right)$ structure, a striped compressed structure and a p(3×3) structure down to potentials close to 0.25 V. There, a very small peak in the CV indicates further Ag deposition and the start of the completion of the 1^st^ Ag monolayer ($p\left(3\times 3\right)$ to (1×1) transition). No well‐resolved atomic‐scale image was observed at the potential near the 2^nd^ Ag UPD peak, only a slight increase in height was observed. Results of surface X‐ray scattering (SXS) shows that the Ag completes a monolayer at the potentials after the 2^nd^ Ag UPD; a bilayer forms at the potential of 3^rd^ Ag UPD.[^6^, ^7^] Although in situ STM and related techniques could reveal the atomic‐level deposition/dissolution process, the Ag UPD process before the completion of Ag monolayer is still not sufficiently understood.

The lateral force microscope (LFM) is used to address frictional forces on the nanometer scale or even in single asperity contact. The strength of interaction between surface and AFM strongly depends on the species adsorbed on the surface. Thus, adsorbed molecules on substrates play a crucial role in adhesion, friction and wear behaviors.[^10^, ^11^, ^12^, ^13^, ^14^, ^15^, ^16^, ^17^, ^18^, ^19^, ^20^, ^21^, ^22^] Interestingly, molecular dynamics(MD) simulation demonstrated that for given external conditions, such as normal load, temperature and adsorbate surface coverage, the observed regime of friction is determined by the strength of the adsorbate‐substrate interaction.[^23^, ^24^, ^25^]

Ionic liquids (ILs) form layered structures at electrode surfaces, as revealed by tip approach curves; friction measurements indicate that the outer layers are penetrated by the tip and only the boundary ion layers, which are in touch with the surface, are closely related with the friction behavior that can be modified by the applied potential.[^16^, ^17^, ^18^, ^22^] Furthermore, smooth layers lead to lower friction values than those with a larger corrugation. In aqueous electrolytes, the surface of Au(111) can be reversibly modified by anions and cations. Our group also has studied the influence of several adlayers on Au(111) on friction force under electrochemical condition.[^11^, ^12^, ^13^, ^14^] For sulfate ions on Au(111) the friction starts to increase at the potential where the lifting of reconstruction of Au(111) occurs and continuously increases with increasing sulfate coverage until the potential arrives at the spike, which indicates the transition of disordered to ordered sulfate adlayer.^[12]^ For low normal loads, friction decreases slightly upon ordering, whereas for high normal load ordering rather leads to a further friction increase. In the case of Cu UPD in sulfuric acid solution, we observed that when the potential approaches the onset of 1^st^ Cu UPD, COF shows the lowest value, resulting from the decreasing anion coverage. Negative of the 1^st^ Cu UPD peak, the copper forms a $\left(\sqrt{3}\times \sqrt{3}R30^{\circ }\right)$ structure with 2/3 coverage, resulting in the increase of the COF. When a Cu monolayer is completed, sulfate ions are adsorbed on Cu(111) and the interaction between sulfate ions and copper substrate leads to the double slip on relatively high loads. Atomic stick slip, which for an electrode surface had first been observed by Labuda et al.,^[26]^ has also be observed at a regularly stepped Au(665) electrode.^[27]^ Friction considerably increases, and stick‐slip becomes irregular upon oxidation of a Au(111) surface.^[28]^

In this study, we investigate the dependence of friction force on applied potentials during Ag UPD on Au(111) in sulfuric acid. On the basis of what is already known from earlier results, it was our aim to understand the influence of adsorbed anions and the coverage of silver during Ag UPD and thus to substantiate and generalize our previous findings on the effects of adsorbates and possible tip penetration.

## 2 Material and Methods

A disc type of Au(111) single crystal(diameter: 10 mm and thickness: 3 mm) purchased from MaTecK GmbH was used as working electrode. It was annealed by flame and cooled down above water purged with Ar to get clean surface. Gold and silver wires were used as counter and reference electrodes, respectively. For electrolytes, 1 mM Ag_2_SO_4_ were dissolved in 0.05 M H_2_SO_4_.

Lateral (frictional) force measurements were performed using Agilent 5500 AFM, combined with glass chamber. Silicon tips (PPP‐FM and and PPP‐CONTSC, NANOSENSORS, tip radius <10 nm) were used and the spring constants were determined to be 0.95±0.05 N/m (PPP‐FM, ‘hard’) and 0.1±0.05 N/m (PPP‐CONTSC, ‘soft’). Normal and torsional resonance frequency of AFM tips were measured by AFM (Agilent 5500/AC mode). Q factor for theses resonance frequencies was obtained using the equation of simple harmonic oscillation (SHO). Normal and torsional force constants were calculated using the Sader method[^29^, ^30^] and the lateral spring constant was obtained by dividing torsional spring constant with the square of the tip height. A homemade AFM cell was used, which contains a three‐electrode assembly and Ar was purged through the glass chamber. The lateral force vs. normal load and potential curves were measured with a scan size of 20×20 nm^2^ and a scan rate of 0.47 nm/s.

When the AFM tip scans the surface, friction (F) makes it twist, which we obtain as the deflection of the laser beam. The sign of the deflection depends on the scan direction. In addition, the deflection of the laser is caused by topography (T) (e. g. steps on the surface), this effect does not depend on scan direction. Thus, the forward image and backward image contain [T+F] and [T−F], respectively. Friction force data was obtained by subtracting the backward data from the forward data and dividing it by 2 to eliminate topographic effects.^[31]^

## 3 Results and Discussion

### 3.1 Cyclic Voltammetry (CV)

**Figure 1:**
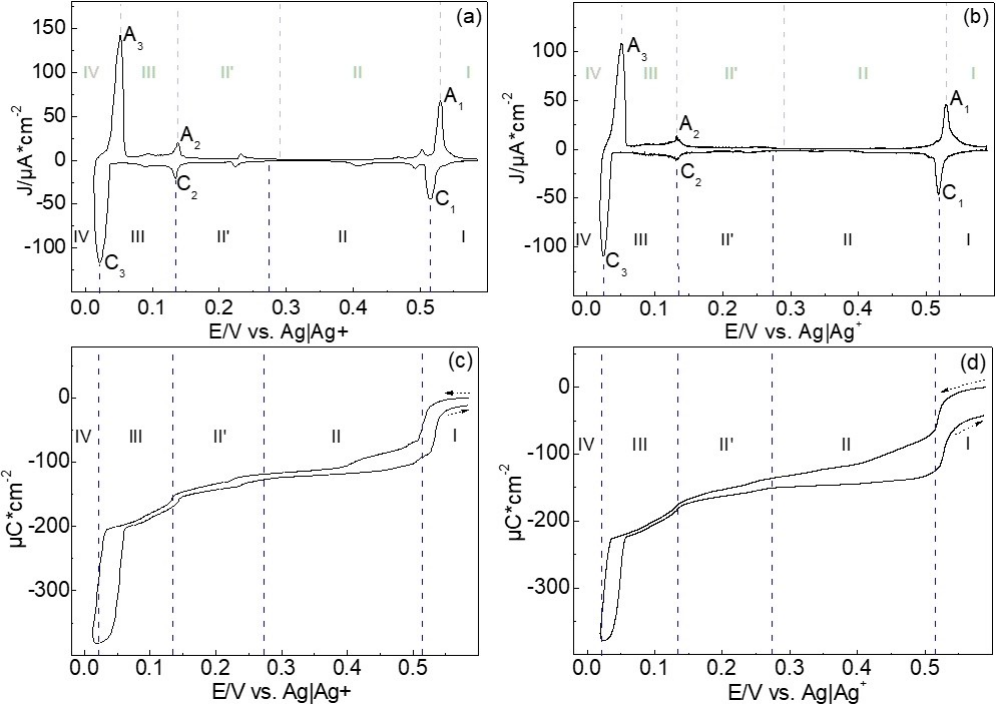
Cyclic voltammetry and coulometric curve in (a) and (c) H‐cell and (b) and (d) AFM‐cell for the Au(111) in 1 mM Ag_2_SO_4_/0.1 M H_2_SO_4_. The sweep rate is 10 mV/s.

Figure 1(a) shows the cyclic voltammetry obtained in H‐cell filled with 1 mM Ag_2_SO_4_/0.05 M H_2_SO_4_. Three major peaks, C_1_, C_2_, and C_3_, with a number of small peaks corresponding to the Ag UPD were observed in agreement with previous reports.[^1^, ^2^, ^3^, ^4^, ^5^] In the AFM‐cell resolution is lower and only three major peaks were visible (Figure 1b). In order to understand the charges associated with peaks, we vary the potential regions (I, II, II', III, and IV) referring to three major peaks. Region II' corresponds to the potential range where Gewirth et al. observed no atomic structure due to the some type of rapid exchange process with the electrolyte.^[4]^ The charge densities corresponding to the first (II), second (II'+III), and third (IV) UPD peaks/regions in H‐cell are 65 (C_1_)+51, 34+57, and 178 μC/cm^2^, respectively.

Regarding the charge density and the results of surface X‐ray scattering (SXS),[^6^, ^7^] silver completes a monolayer at slightly negative potential of C_2_ and peak C_3_ is related with a bilayer formation. The total charge for completion of the first monolayer (region II–III, 207 μC/cm^2^) is less than the theoretical charge (222 μC/cm^2^) which is explained by the shift of the pzc upon deposition of Ag, which on Ag is 1 V more negative than that of Au, and the corresponding anionic charge flow.^[3]^ The charge flow in the broad region II and II' may indicate that the silver is continuously deposited on the surface as shown in results of the electrochemical quartz crystal microbalance (EQCM).^[32]^

### 3.2 Friction Measurement

#### 3.2.1 Atomic Corrugation in Friction

**Figure 2:**
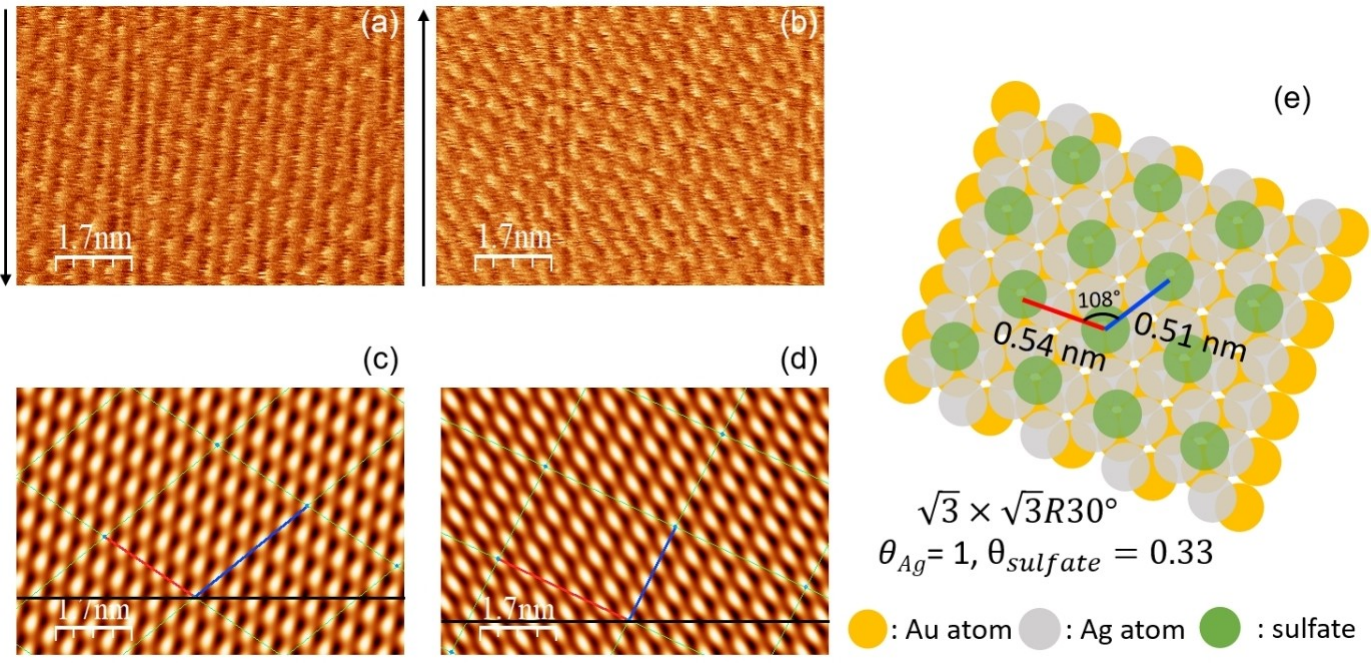
Lateral map at 0.02 V (vs. Ag/Ag+) in cathodic sweep during (a) downward scan and (b) upward scan. Arrow indicates scan direction. (c) and (d) are the lattice images after FFT filtering of (a) and (b), respectively. (e) is the illustration of the real lattice with lattice parameters after the correction of thermal drift. PPP‐FM was used (k_N_=0.95±0.05 N/m). Applied normal load is 8 nN.

**Figure 3:**
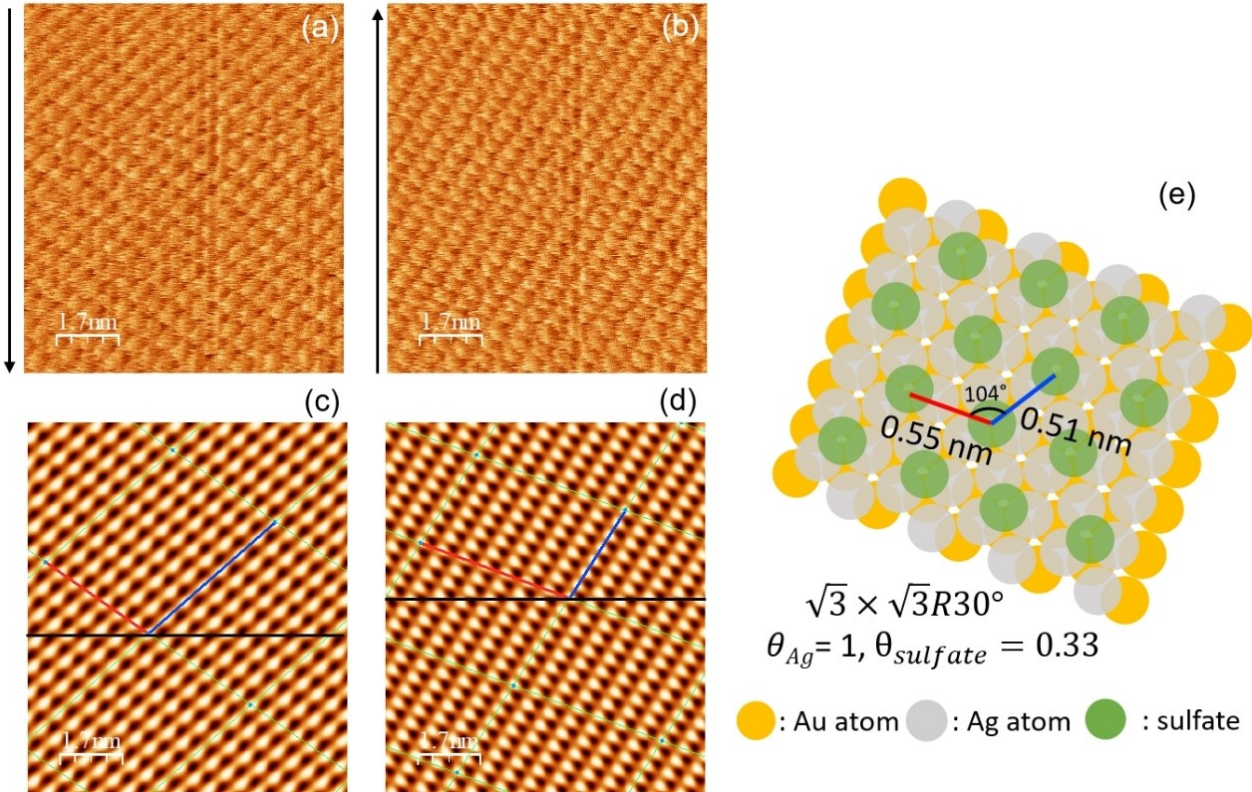
Lateral map at 0.09 V (vs. Ag/Ag+) in anodic sweep during (a) downward scan and (b) upward scan. Black arrow indicates scan direction. (c) and (d) are the lattice images after FFT filtering of (a) and (b), respectively. (e) is the illustration of the real lattice with lattice parameters after the correction of thermal drift. PPP‐FM was used (k_N_=0.95±0.05 N/m). Applied normal load is 8 nN.

We investigated structures depending on potentials using atomic corrugation in friction images to get the information for the coverage of adsorbates (sulfate and Ag). To get clear lattice image we used fast Fourier transform (FFT) filtering and corrected for thermal drift.^[33]^ As shown in Figure S1, we observed the well‐known $\left(\sqrt{3}\times \sqrt{7}R19.1^{\circ }\right)$ structure of the ordered sulfate/bisulfate layer on Au(111) at 0.57 V. At potentials between the 1^st^ and 2^nd^ Ag UPD peaks (region II and II’), it was not easy to get atomically well resolved images, which may be due to the fact that several structures seem to coexist in this potential region and their instability.[^1^, ^2^, ^3^, ^4^, ^5^] Sometimes, we observed the $\left(\sqrt{3}\times \sqrt{3}R30^{\circ }\right)$ structure at 0.33 V as shown in Figure S2. At 0.0 V, where we assume that silver completes a Ag bilayer, we easily observed the $\left(\sqrt{3}\times \sqrt{3}\right)R30^{\circ }$ structure as shown in Figure 2. In anodic sweep, at slightly positive of peak A_3_, 0.09 V, where a monolayer exists after the dissolution of a Ag bilayer, the $\left(\sqrt{3}\times \sqrt{3}\right)R30^{\circ }$ structure was also observed (Figure 3). It supports the previous results of EQCM that the amount of adsorbed sulfate is rather constant during the Ag UPD (region II–IV).^[32]^

#### 3.2.2 Friction Force Dependence on Potential

**Figure 4:**
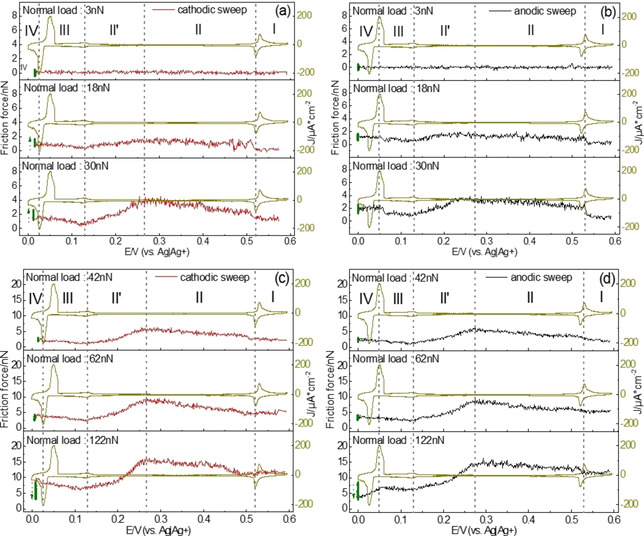
Friction force on potential at fixed normal loads during (a) and (c) cathodic sweep and (b) and (d) anodic sweep. PPP‐FM was used (k_N_=0.95±0.05 N/m). The scan rate and size of AFM images were 0.47 nm/s and 20X20 nm^2^, respectively.

Figure 4 shows the dependence of friction on potential. In this measurement, the sweep rate of CV was set as 14 mV/s, which fits the speed for the completion of a lateral force map. When a potential arrives at about 0.02 V in cathodic sweep, one friction image was recorded at 0.02 V for the complete Ag bilayer before starting the anodic sweep; the corresponding friction is marked as green dots in Figure 4.

When the applied normal load is below 5 nN, no friction change is observed. Above this minimal load, the friction shows strong dependence on the coverage of adsorbates. When the applied potential is more positive than the 1^st^ Ag UPD peak, region I, the sulfate/bisulfates are adsorbed on Au(111) and form the $\left(\sqrt{3}\times \sqrt{7}\right)R19.1^{\circ }$ structure ($\theta_{sulfate}=0.2)$ . Friction therefore is dominated by the interaction between AFM tip and the sulfate layer.

Slightly negative of the 1^st^ Ag UPD peak, the deposition of silver causes a sudden increase in friction. Moreover, friction continuously increases with decreasing potential in region II. In this region, Kolb et al. observed three different structures by STM (a $\left(\sqrt{3}\times \sqrt{3}\right)R30^{\circ }$ , compressed $\left(\sqrt{3}\times \sqrt{3}\right)R30^{\circ }$ , and, predominantly, a p(3×3) structure) in region II,^[3]^ whereas Itaya[^1^, ^2^] mainly found the $\left(\sqrt{3}\times \sqrt{3}\right)R30^{\circ }$ structure and Gewirth,[^4^, ^5^] by AFM, only the p(3×3) structure. Since we observed the $\left(\sqrt{3}\times \sqrt{3}\right)R30^{\circ }$ structure at 0.33 V, 0.09 V, and 0.0 V, we speculate that sulfate constantly forms this structure after the 1^st^ Ag UPD peak, at least in some domains, independent of the overall coverage of silver. The deposition of Ag induces a higher coverage by sulfates due to the change of the pzc, thus partially compensating the charge density of the 1^st^ Ag UPD. The transition of sulfate structures from the $\left(\sqrt{3}\times \sqrt{7}\right)R19.1^{\circ }$ to the $\left(\sqrt{3}\times \sqrt{3}\right)R30^{\circ }$ structure leads to a change in coverage of Δ$\theta_{sulfate}$ =0.2–0.33, corresponding to a change of charge density of anions during the 1^st^ Ag UPD of 29 μC/cm^2^ in good agreement with our experimental value of about 24 μC/cm^2^. Thus, depending on imaging conditions, either this sulfate structure is observed or that of the underlying Ag adlattice with a possible p(3×3) structure. A corresponding structure model for the $\left(\sqrt{3}\times \sqrt{3}\right)R30^{\circ }$ structure of sulfate layer on the p(3×3) structure of silver layer is shown in Figure S3. We explain the continuously increasing Ag coverage over the large potential range II and the obvious instability of the structures by the simultaneous change of the pzc with increasing Ag coverage, which is shifting in negative direction; on a rational potential scale (referenced with respect to the pzc), the potential is becoming more positive with increasing Ag coverage. The increase of friction in region II suggests that the adsorbate layers (sulfate and Ag) are highly mobile on the surface. We observed such an increasing high friction on Au(111) in pure sulfuric acid, when with increasing potential the sulfate coverage increases, as long as the adlattice is disordered below the potential of the well‐known spike indicating the phase transition to the ordered $\left(\sqrt{3}\times \sqrt{7}\right)$ structure.^[12]^ Similarly, in 0.4 mM CuSO_4_/0.05 M H_2_SO_4_, friction decreases when sulfate is desorbed upon a potential sweep in negative direction reaching a minimum before Cu sets in.^[13]^ Such a dependence of friction on coverage by a mobile phase is also in agreement with the MD simulation of Ouyang et al..^[25]^ This continuous increase of friction is different from the case of Cu UPD, where friction is independent of potential when the copper forms the immobile honeycomb structure as shown in Figure S4.^[34]^

The sudden increase of friction is delayed at higher normal load of 62 nN and even more 122 nN. This may be due to the AFM tip penetrating the incomplete Ag‐submonolayer at high normal loads and thus pushing away the silver ions near the tip, which locally leads to the negative shift of the potential for the adsorption of silver ions.^[12]^

At 0.27 V friction starts to decrease. This is the potential where the transition to the $\left(1\times 1\right)$ adlayer starts (region II'),^[3]^ because the $\left(3\times 3\right)$ adlayer with a coverage of 0.45 is completed and therefore silver is no longer mobile. After penetration of the sulfate adlayer it pushes the ions on a fairly smooth Ag layer or slides on the layer. Friction further decreases when the potential approaches the second Ag UPD peak at about 0.1 V because the completed $\left(1\times 1\right)$ Ag layer is even smoother.

Negative of peak C_2_ (region III) friction slightly increases again; this is paralleled by a fairly large current the origin of which is not discussed in previous papers.^[3]^ This charging and friction increase might indicate deposition of further, disordered and fluctuating Ag ad‐atoms initializing deposition of the 2^nd^ layer and adsorption of sulfate.

Negative of peak C_3_, where a bilayer forms, friction force changes with time (cf. green dots in Figure. 4). The direction of this change shows a dependence on normal load: At low normal load (10 nN<F_N_<40 nN) friction increases with time; when the normal load exceeds 40 nN, this behavior disappears. Astonishingly, at 122 nN, friction decreases with time. Moreover, in anodic sweep, the friction increases when the potential approaches and passes peak A_3_. On the contrary, at low normal loads (30 nN), friction drops after passing peak A_3_. According to Kolb et al., formation of the 2^nd^ layer in the peak C_3_ does not immediately lead to a smooth surface. Rather, a rough, dynamic 2^nd^ layer only slowly transforms into a complete, smooth 2^nd^ Ag layer after waiting several minutes close to 0 V. It is tempting to assume that the time dependent change of friction at this potential is related to this slow deposition process.

Our results presented above showed that sulfate forms the $(\sqrt{3}\times \sqrt{3)}R30^{\circ }$ structure ($\theta_{sulfate}=0.33$ ) both on the Ag bilayer and the completed Ag–monolayer, i. e. on both sides of peak C_3_/A_3_. However, one may presume that sulfate on the bilayer is more strongly bound, since the pzc of the bilayer can be assumed to be nearly identical to that of bulk Ag whereas that of a monolayer might be up to 200 mV more positive.^[35]^ This could lead to a higher friction for the monolayer once the tip is penetrating the sulfate layer. The very high normal load of 122 nN, on the other hand, might locally prevent deposition of the 2^nd^ adlayer, but why this could lead to a reduced friction remains completely unclear at this point.

#### 3.2.3 Friction Dependence on Normal Load

**Figure 5:**
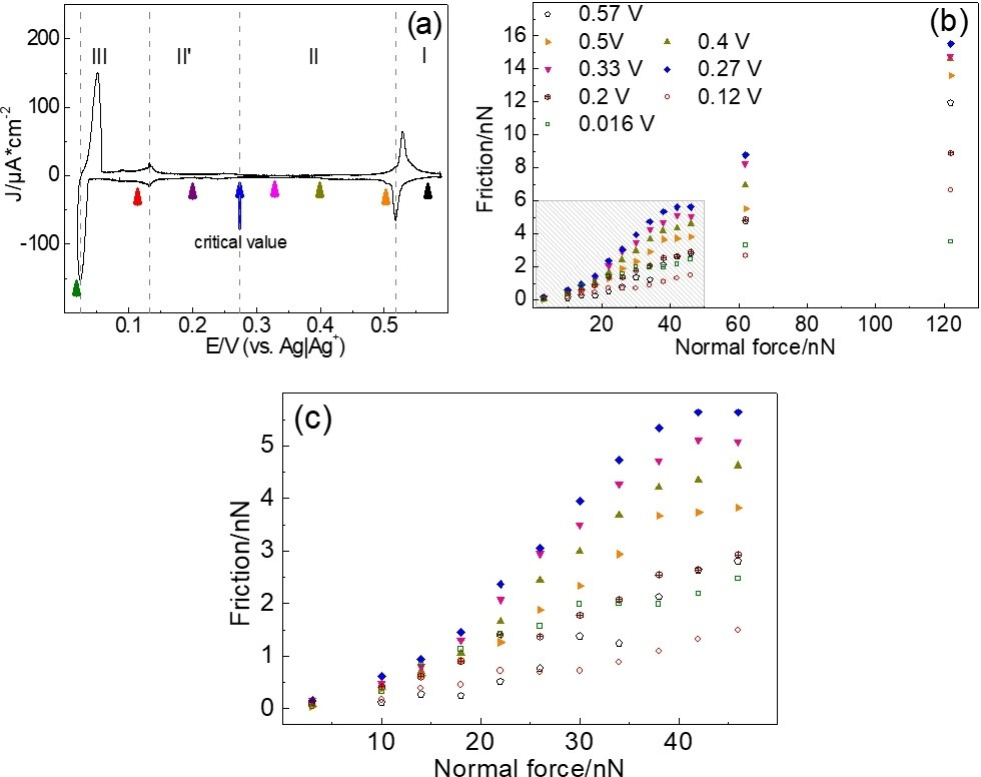
(a) CV obtained in AFM‐cell and (b) data from Figure 4 plotted as function of normal load during Ag UPD. (c) shaded area of (b). Potentials are remarked with same colored arrows in the CV.

The data of Figure 4 (together with additional data) are replotted as a function of load in Figure 5 (also cf. Figure S5). For the friction at 0.016 V, data in the anodic sweep was used because of the time dependence. Interestingly, friction versus load shows at least three different sections or regimes, depending on different extents of penetration. For very low normal loads (F_N_<14 nN), the COF is low for all potentials. We assume that here the tip is sliding above any adsorbate layer. The COF then increases above a critical load, which depends on potential (regime α). We had observed this effect before for the Cu‐upd system and also for sulfate on Au(111) and tentatively ascribed it to penetration of the tip into the sulfate adlayer,[^12^, ^13^] where more and more adsorbate species interact with the tip and have to be pushed away. For higher normal loads, as suggested also by the modelling in,^[25]^ the adsorbate is completely squeezed out from the confined region underneath the tip, “and the number of particles in contact with the tip levels off. In this second regime, the main contribution to the frictional energy dissipation comes from the adsorbates pushed by the tip along the surface. This contribution depends weakly on the normal load, leading to a low friction coefficient”^[25]^ (the plateau of regime β). Thereafter, in regime γ friction increases linearly with normal load (Amontons’ behavior); this friction force adds to the force necessary for displacement of the adsorbate species of regime β. Since the tip penetrates into the adsorbate layer, we presume that the interaction between adsorbate and substrate is an important factor for the COF and additionally, the interaction between tip and substrate (e. g. hydrogen bond, covalent bond) might have influence on it.^[36]^

This behavior shall now be discussed separately for the different potential regions. At 0.57 V where the sulfate forms the $\left(\sqrt{3}\times \sqrt{7}\right)R19.1^{\circ }$ structure, the friction increases with increasing normal loads at low normal loads (15 nN<F_N_<30 nN) and high normal load (38 nN<F_N_), indicating regime α and γ, respectively. The COFs for regime α and γ are 0.09 and 0.12, respectively. Before the transition from regime α to regime γ, the friction shows a plateau, regime β. We may infer that in accordance with the modelling of ref^[25]^ with increasing normal loads the tip starts to penetrate into sulfate layer resulting in the increase of friction (regime α). At certain normal load the tip penetrates into sulfate layer and pushes away the adsorbed sulfates (regime β), which does not depend much on load. Considering COFs at high normal load (regime γ), where presumably the tip is in direct contact with the Au substrate, we suggest that the multiple interactions between tip, sulfate, and the surface of Au(111) have an effect on this regime.

At potentials in region II, where the adsorbates (sulfate and Ag) form instable structures, the friction shows a dramatic increase with increasing normal loads in regime α (18 nN<FN<38 nN). Moreover, the COF increases with decreasing potentials (0.14 (0.5 V), 0.16 (0.4 V), 0.17 (0.33 V), and 0.2 (0.27 V)). It resembles the friction results for Au(111) in 0.1 M H_2_SO_4_ from Hausen et al. where the COF increases with increasing the coverage of sulfate when the sulfate is disordered (Figure S6).^[12]^ Considering the instable structure of adsorbates including frizzy nature of monoatomic steps on Ag(111) surfaces[^37^, ^38^] and the continuously increasing Ag coverage, we envisage two possibilities: Either the tip penetrates only the sulfate adlayer. Continuous displacement and pushing sulfate upon scanning may require more energy on a disordered Ag layer, which strengthens the resistance against the sliding of the tip.^[28]^ Or, the tip penetrates into the sulfate layer as well as the Ag layer. At this point, we cannot really separate the contribution of sulfate and Ag layers in regime α; considering the atomic stick‐slip at 0.27 V (Figure S7), the penetration into Ag layer might be dominant influence. The friction increase with decreasing potential is due to the increasing number of Ag atoms which have to be moved or which are hindering the movement of sulfate in front of the tip. In regime β, the friction shows a clear plateau, as discussed above. At high normal loads (regime γ, 46 nN<F_N_), the friction increases linearly and the COF is constantly 0.12, resulting from the multiple interactions between tip, adsorbates (sulfate and Ag), and the surface of Au(111).

At 0.2 V (potential region II’), the COFs (0.08) in regime α and γ are lower than all the values in region II, whereas the extensions of regime α and γ are same as for region II. This indicates that beyond a critical value for the Ag coverage (E<0.27 V), where according to Ref. [3] the amount of deposited Ag exceeds the coverage of 0.45 corresponding to the p(3×3) Ag adlayer and where thus the transition to (1×1) adlayer starts, the tip only penetrates into sulfate layer. Therefore, the tip is sliding on a smooth, probably well‐ordered p(3×3) Ag adlayer and therefore the COF is reduced.

At 0.12 V (region III), where we assume the formation of a complete Ag monolayer, the COF in regime α (10 nN<F_N_<24 nN) and in regime γ (30 nN<F_N_) is further decreased to 0.04 and 0.07, respectively. This may be due to the much smoother Ag(1×1) adlayer, on which the tip is sliding after displacement of sulfate. At 0.016 V where a bilayer completes, the COF in regime α (10 nN<F_N_<30 nN) and in regime γ (38 nN<F_N_) are 0.07 and 0.02, respectively. Considering the ordered structure of sulfate ($\left(\sqrt{3}\times \sqrt{3}\right)R30^{\circ }$ ), the slight increase of COF in regime α is reasonable as discussed above, whereas an explanation of the independence of friction from normal load in regime γ might indicate the penetration of the tip into the 2^nd^ (top) Ag‐adlayer (as discussed before for sulfate). However, this as well as the observed time dependence in this potential region needs further consideration. In a similar way we can interpret the results of Ref. [12] (together with Figure S6): the higher the sulfate coverage, the larger the normal load for penetration of the tip has to be. Upon ordering, this necessary load is further increased, and therefore ordering leads to increased friction for high normal loads as opposed to low normal loads, for which ordering seems to lead to an easier sliding of the tip above the sulfate.

We should add that at the high normal loads used in regime γ one might expect the occurrence of wear. We did not find any indication for that. We had observed previously that for the Au(111)/Cu UPD system local alloy formation induced by wear was only observed when a Pt covered tip was used and the normal load was 1 μN^[39]^ or, for several UPD systems, when scanning with an STM tip under conditions where a quantum nano‐contact is formed.[^40^, ^41^, ^42^] But it is also clear that if the tip penetrates the Ag adlayer this is already the first stage of wear; tip induced displacement or local dissolution of Ag, however, does not lead to a structural change, this kind of wear is self‐healing.

## 4 Conclusions

Friction force images show clearly resolved structures for sulfate adsorbed on a densely packed Au(111) substrate positive of Ag UPD $\left(\sqrt{3}\times \sqrt{7}R19.1^{\circ }\right)$ ($\theta_{sulfate}=0.2)$ , and also on the complete monolayer or complete bilayer of Ag on Au(111) $\left(\sqrt{3}\times \sqrt{3}R30^{\circ }\right)$ ($\theta_{sulfate}=0.33$ ). However, in the whole potential range where the Ag monolayer is not completed a clear structure could not reproducibly observed, despite of the observed atomic stick‐slip. This is certainly due to the high mobility of Ag in such an open structure and also the interaction with the tip. The dependence of friction on potential reflects the complexity of this UPD system. Friction increases with coverage of Ag until the coverage arrives at a critical value (0.27 V) and then decreases again when due to more Ag deposition the formation of the monolayer is initiated and probably the mobility of Ag atoms on the surface is reduced. The friction forces plotted as function of load demonstrate once again a non‐monotonic dependence of the friction force on the coverage of adsorbates (sulfate and Ag). In general, the behavior of friction on normal load can be divided into three regimes; in the process of penetration into adsorbate layer (regime α), after the penetration (regime β), and interaction with metal substrates (Au and Ag) (regime γ). The coverage and stability of adsorbates are determining factors for the COF in regime α. In general, the higher the coverage, the larger the normal load necessary for complete penetration of the tip seems to be. In regime β, the tip pushes away a load independent (but potential dependent) number of adsorbed species; it would be interesting to elucidate the dependence of this behavior on surface mobility of an adsorbate in further studies. Thus, the change of friction on normal load is negligible in this regime. At high normal load (regime γ), additional interaction between tip and substrate results in linear increase of friction on normal load. Atomic‐scale friction force measurements under electrochemical condition thus provide a way to understand the interaction between the surface and a single asperity. Further work would be necessary to elucidate and separate the effects of surface charge and double layer, which are screened in the present work by the strong adsorption (including their partial discharge) of silver and sulfate.

## Conflict of interest

The authors declare no conflict of interest.

## Supporting information

As a service to our authors and readers, this journal provides supporting information supplied by the authors. Such materials are peer reviewed and may be re‐organized for online delivery, but are not copy‐edited or typeset. Technical support issues arising from supporting information (other than missing files) should be addressed to the authors.

Supplementary
