# Supplementary material for: In situ friction study of Ag Underpotential deposition (UPD) on Au(111) in aqueous electrolyte
Source: Chemphyschem. 2021 May 4;22(10):952–9. doi: 10.1002/cphc.202100130 (PMC8252634; doi:10.1002/cphc.202100130)
Supplement: Supplementary file 1 — Supplementary [file CPHC-22-952-s001.pdf]

ChemPhysChem

Supporting Information

In situ friction study of Ag Underpotential deposition (UPD) on Au(111) in aqueous electrolyte

Inhee Park and H. Baltruschat*

Supporting Information

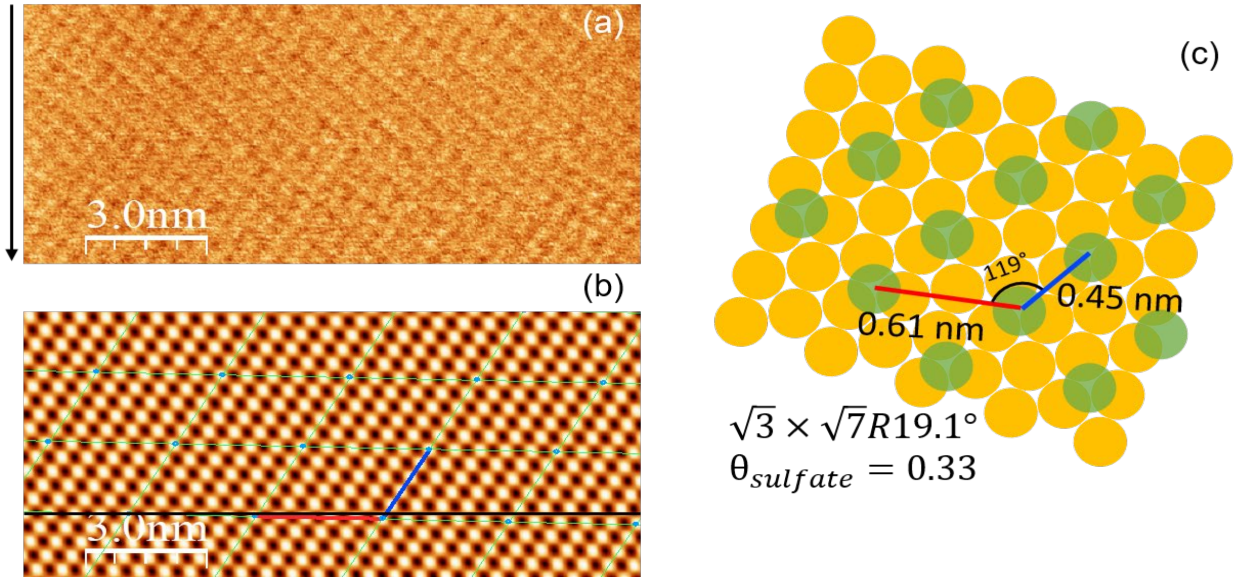

Figure S1 (a) Lateral map at 0.57V (vs. Ag/Ag+) during downward scan. Black arrow indicates scan direction. (b) is the lattice image after FFT filtering of (a). (c) is the illustration of the real lattice with lattice parameters after the correction of thermal drift. PPP-FM was used (k_N = 0.95±0.05 N/m). The scan rate and size of (a) is 0.47 nm/s and 20X20 nm^2. Applied normal load is 14 nN.

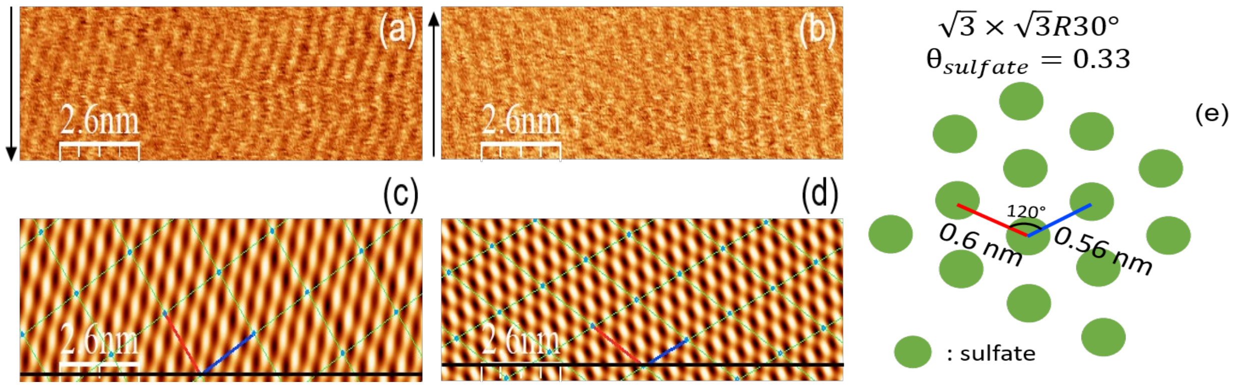

Figure S2 Lateral map at 0.33V (vs. Ag/Ag+) in cathodic sweep during (a) downward scan and (b) upward scan. Black arrow indicates scan direction. (c) and (d) are the lattice images after FFT filtering of (a) and (b), respectively. (e) is the illustration of the real lattice with lattice parameters after the correction of thermal drift. PPP-FM was used (k_N = 0.95±0.05 N/m). Applied normal load is 16 nN

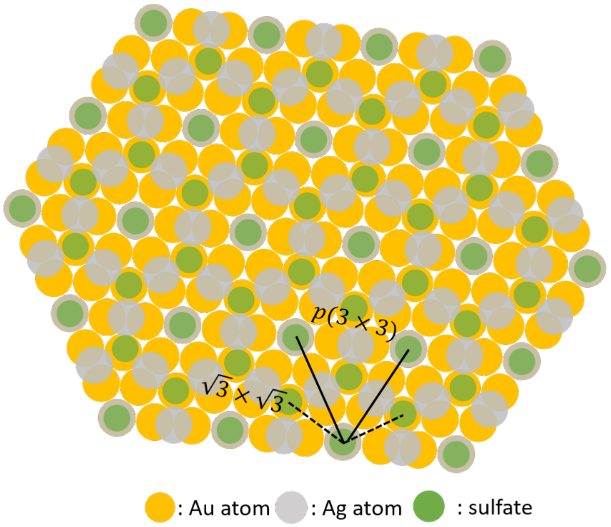

Figure S3 Schematic representation of the $p(3 \times 3)$ structure for the silver and the $(\sqrt{3} \times \sqrt{3})R30°$ structure for the sulfate.

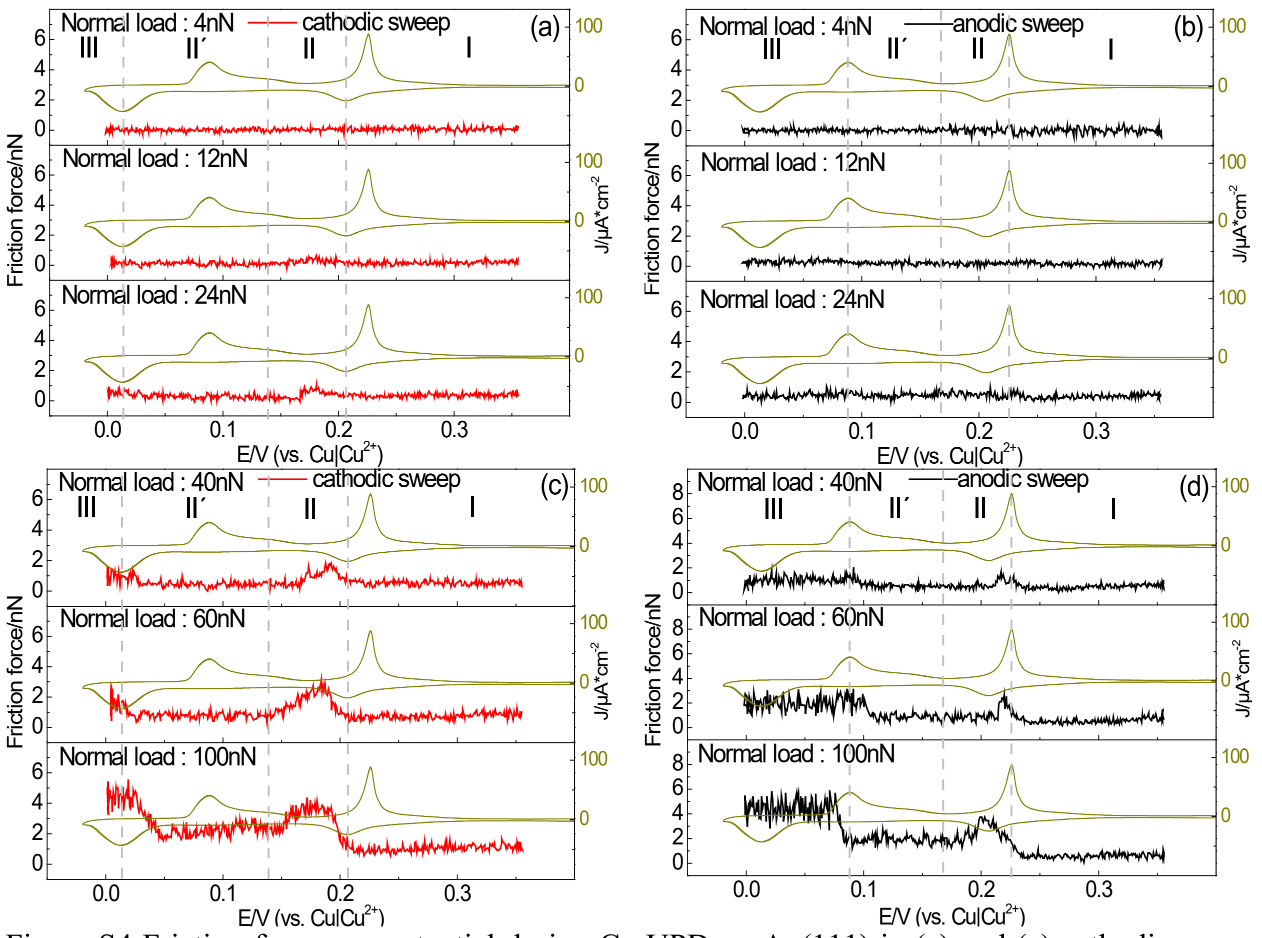

Figure S4 Friction forces on potential during Cu UPD on Au(111) in (a) and (c) cathodic sweep and (b) and (d) anodic sweep. PPP-FM was used (k_N = 0.95±0.05 N/m). The scan rate and size of AFM images were 0.47nm/s and 20X20nm², respectively.

Figure S4 shows the friction results on potential in Cu^{2+} containing electrolyte. These data reproduce the data of ref. [1], but are more detailed. In region I, with decreasing the potential (cathodic sweep) disordered sulfate is desorbed, which resulting in the decrease of friction. It is in good agreement that the pure Au(111) (E = 0.22 V) shows the lowest friction [1]. Further decrease of potential causes the deposition of Cu on Au(111), which leads to the increase of friction (region II). Thus, the friction increases dramatically due to the increasing the coverage of Cu. Whenever the copper forms honeycomb structure ($(\sqrt{3}\times\sqrt{3})R30°$ and $\theta_{Cu} = 2/3$), friction is independent of potential (region II′).

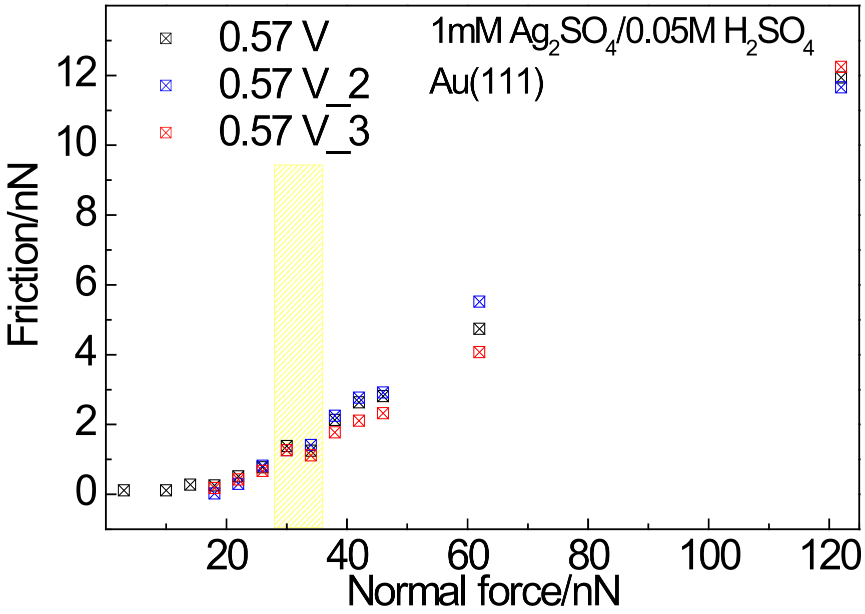

Figure S 5 Data as in Figure 5, plotted as function of normal load at 0.57 V. Three different areas in lateral map were chosen to make clear the behavior of plateau (shaded area) indicating the regime β.

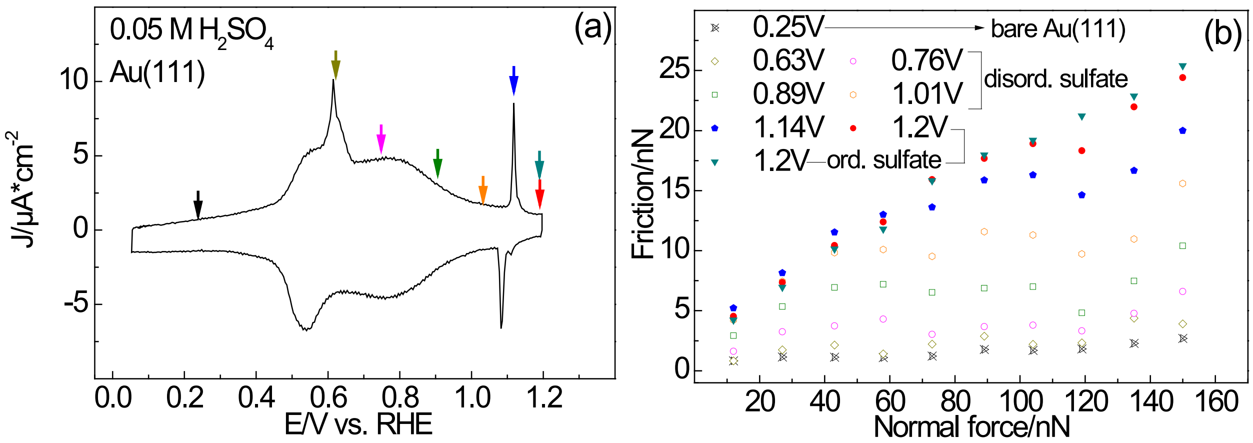

Figure S 6 Paper from Hausen et al. [2] CV obtained on Au(111) in 0.05 M H_2SO_4. (b) Friction as function of potential was replotted as function of normal load. The potential stopped at 1.20; therefore, data at 1.20 V represent changes with time.

Figure S6 shows the results replotted as function of normal load from the data in the paper from Hausen et al [2]. At 0.63 V where sulfate is adsorbed on Au(111) and lifting the reconstruction of Au(111), the friction on normal load still behaves similarly as observed on bare Au(111) (0.25 V). At 0.76 V, at low normal load ($F_N < 60$ nN), with increasing normal load friction increases slightly. It indicates that in regime α ($F_N < 60$ nN) friction is due to the penetration into adsorbed sulfate layer. The COF in regime α is 0.06. At high normal load (120 nN < F_N) friction increases again with increasing normal load. It indicates regime γ where the interaction with gold surface is dominating. Between regime α and γ, the friction shows plateau and it indicates regime β. When the potential approaches to 0.89 V, the COF in regime α increases to 0.12. It indicates that the COF in regime α is closely related with the coverage of sulfate if it is disordered. When the sulfate is ordered (1.20 V), the range of regime β seems to be reduced meaning that the dependence of friction on normal load is rather linear.

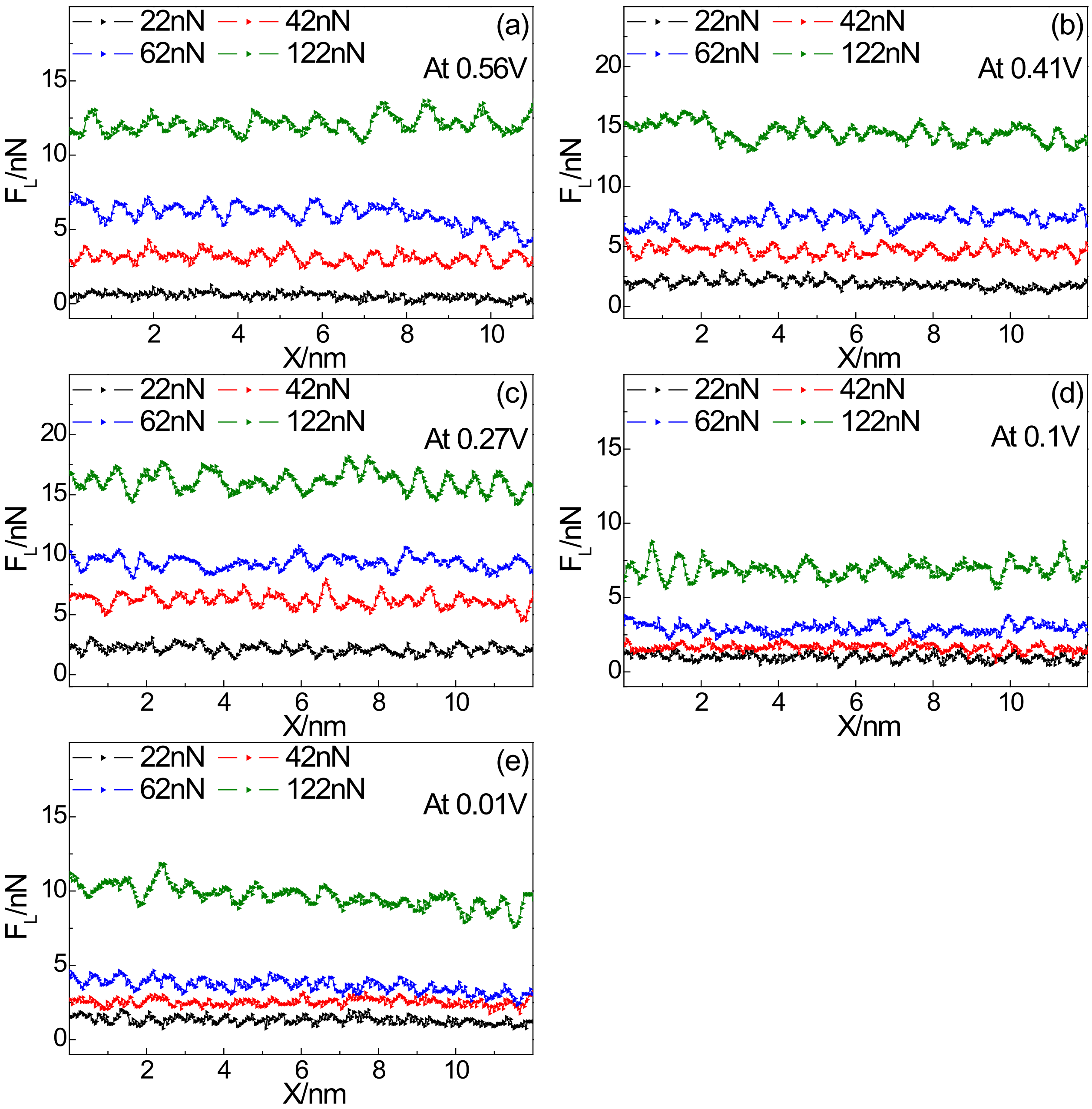

Figure S 7 Atomic stick-slip during Ag UPD at (a) 0.56 V, (b) 0.41V, (c) 0.27V, (d) 0.1V, and (d) 0.01V on Au(111) at normal load 22, 42, 62, and 122nN. PPP-FM (k_N = 0.95±0.05 N/m) was used.

Figure S7 shows the atomic stick-slip depending on potential. At 0.56 V where the adsorbed sulfate forms the $(\sqrt{3} \times \sqrt{7})R19.1°$ structure, no clear atomic stick-slip is observed at 22nN but at the normal load above 40nN, identical atomic stick-slips are obtained. The distance between sticks is about 0.7 nm. We assume that the adsorbed sulfate forms instable structure at 0.41 V and 0.27 V (potential region II), which might cause the atomic stick-slip at lower normal load. The distance

between sticks is about 0.7 nm. The atomic stick-slip at 0.1 V and 0.01 V look similar and it might result from the same coverage of Ag ($\theta_{Ag} = 1$).

[1] N. Podgaynyy, S. Wezisla, C. Molls, H. Baltruschat *BEILSTEIN JOURNAL OF NANOTECHNOLOGY*. **2015**, *6*, 820-830.
[2] F. Hausen, M. Nielinger, S. Ernst, H. Baltruschat *Electrochimica Acta*. **2008**, *53*, 6058-6063.
